# Medical Association of Georgia

**Published:** 1885-02

**Authors:** 


### THE MEDICAL ASSOCIATION OF GEORGIA.

This Association ought to be the pride of every physician in the State. Judging, however, from the number of names on the roll, one would very naturally conclude—and justly so—that very little interest is felt in it by the majority of the physicians in the State. This want of interest is nowhere more manifest, with possibly one or two exceptions, than among the officers of the Association.

Nothing, so far as we are aware, is being done to bring the Association to the attention of the many physicians in the State not members, in order that they might become members. The Association ought to be an influential body, and would be if only the proper interest was taken in it by the officers as well as the individual members.

In order to ascertain just how little influence the Association has, we have but to appear before a legislative committee and ask for any measure looking to the interest of the profession in the State, we will then find that we have no influence at all. If, however, the Association had one thousand or fifteen hundred members, instead of two hundred and fifty-six, as it now has, it would then command respect at the hands of the law-makers.

There are over two thousand physicians in Georgia to-day; a vast majority of that number could not tell you, if asked, whether there positively was a State Association or not, and fewer still could tell you who the President of the Association was, and when the annual meetings were held. Of these two thousand physicians, at least two-thirds of them ought to be members of the State Association, and we believe would be if the proper methods were adopted to get them to become such.

Alabama has the best organized State Association of any State in the Union, and it was only obtained after a great deal of hard work. It would have been in the condition of our own Association to-day but for the enterprise, energy and perseverance of a few of her best men.

What Alabama has accomplished Georgia can accomplish, if only the same zeal and energy is manifested by the officers and members of her Association as was manifested by the men in the Alabama Association.

We would suggest that a call be issued to every physician in the State urging the importance of thorough organization, and inviting them to attend the coming meeting of the Association in Savannah, and to unite themselves with it.

